# The promises and challenges of pre-exposure prophylaxis as part of the emerging paradigm of combination HIV prevention

**DOI:** 10.7448/IAS.18.4.19949

**Published:** 2015-07-20

**Authors:** Carlos F Cáceres, Florence Koechlin, Pedro Goicochea, Papa-Salif Sow, Kevin R O'Reilly, Kenneth H Mayer, Peter Godfrey-Faussett

**Affiliations:** 1 Center for Interdisciplinary Studies in Sexuality, AIDS and Society, Universidad Peruana Cayetano Heredia, Lima, Peru; 2 Network for Multidisciplinary Studies in ARV-Based HIV Prevention (NEMUS), Lima, Peru; 3 HIV Department, World Health Organization, Geneva, Switzerland; 4 Center for AIDS Prevention Studies, University of California – San Francisco, San Francisco, CA, USA; 5 The Bill and Melinda Gates Foundation, Seattle, WA, USA; 6 The Fenway Institute, Fenway Health, Boston, MA, USA; 7 Joint United Nations Programme on HIV/AIDS, Geneva, Switzerland

**Keywords:** HIV prevention, pre-exposure prophylaxis, public health, health policy, antiretrovirals

## Abstract

**Introduction:**

Towards the end of the twentieth century, significant success was achieved in reducing incidence in several global HIV epidemics through ongoing prevention strategies. However, further progress in risk reduction was uncertain. For one thing, it was clear that social vulnerability had to be addressed, through research on interventions addressing health systems and other structural barriers. As soon as antiretroviral treatment became available, researchers started to conceive that antiretrovirals might play a role in decreasing either susceptibility in uninfected people or infectiousness among people living with HIV. In this paper we focus on the origin, present status, and potential contribution of pre-exposure prophylaxis (PrEP) within the combination HIV prevention framework.

**Discussion:**

After a phase of controversy, PrEP efficacy trials took off. By 2015, daily oral PrEP, using tenofovir alone or in combination with emtricitabine, has been proven efficacious, though efficacy seems heavily contingent upon adherence to pill uptake. Initial demonstration projects after release of efficacy results have shown that PrEP can be implemented in real settings and adherence can be high, leading to high effectiveness. Despite its substantial potential, beliefs persist about unfeasibility in real-life settings due to stigma, cost, adherence, and potential risk compensation barriers.

**Conclusions:**

The strategic synergy of behavioural change communication, biomedical strategies (including PrEP), and structural programmes is providing the basis for the combination HIV prevention framework. If PrEP is to ever become a key component of that framework, several negative beliefs must be confronted based on emerging evidence; moreover, research gaps regarding PrEP implementation must be filled, and appropriate prioritization strategies must be set up. Those challenges are significant, proportional to the impact that PrEP implementation may have in the global response to HIV.

## Introduction

By the end of the twentieth century, two decades after the HIV epidemic became visible, substantial success in slowing down HIV transmission had been achieved with ongoing prevention strategies, but it seemed far from stopping epidemic growth [1, 2]. Conversely, the use of combined antiretroviral treatment (ART) to prevent disease progression [3] and the development of effective regimes to prevent mother-to-child transmission (PMTCT) [4] were major steps in the HIV response.

As a corollary to “highly active antiretroviral treatment,” people discussed the possibility of “highly active prevention” [5]. The development of effective antiretrovirals (ARVs) led, from the start, to conceiving of biomedical prevention tools to decrease either susceptibility in uninfected people or infectiousness among people living with HIV (PLH). Given the lack of an effective HIV vaccine, key trials addressed several testable strategies that applied biomedical principles to HIV prevention, including sexually transmitted infection (STI) control, male medical circumcision, and the oral or topical use of ARV drugs to reduce susceptibility among the uninfected [6–14], as well as earlier ART initiation among positives to reduce infectivity [15]. Over a decade later, several new prevention strategies have been proven efficacious, and others are very promising [16, 17]. Among newly available technologies, evidence of real-life effectiveness is quickly accumulating for oral PrEP.

Simultaneously, in line with the WHO Social Determinants of Health approach [18], HIV risk is now understood as resulting from individual, interpersonal, community-level, and social-structural factors [19, 20]. In response to those, “structural interventions” targeting legal, institutional, social, cultural, and economic determinants of HIV vulnerability are considered essential to the HIV response [21].

This new environment of bio-behavioural, individual-level interventions, along with appropriate social-structural strategies, is consolidating a framework for combination prevention [22–24]. In this paper, we focus on the origin, present status, and potential contribution of PrEP within the combination HIV prevention framework.

## Discussion

### Pharmacologic prophylaxis in public health and the concept of HIV PrEP

Over the past few decades, the use of specific medications to prevent clinical conditions has become standard practice in preventive medicine [25–32]. Within the HIV field, secondary prophylaxis against *Pneumocystis carinii* pneumonia with cotrimoxazole was introduced among PLH [33]. After several evolving regimes, in 2013 WHO recommended that all women living with HIV found to be pregnant should start regular ART, whereas newborns should receive a six-week ARV course [34]. Over time, PMTCT approaches and successes have played a key role in shaping the initial thinking about using PrEP to prevent sexually acquired HIV infections.

### Early controversies around oral PrEP clinical trials

An overview of the complex history of ideas and research that contributed to the development of PrEP is relevant as it may, in part, explain some remaining controversies [35]. After animal studies demonstrated the protective effects of pre-exposure ARV use and successful early phase human studies demonstrated safety [36–38], the planning of phase III trials to assess the efficacy of oral PrEP using tenofovir (tenofovir disoproxil fumarate (TDF), Viread^®^) generated interest among HIV prevention scientists, but also some concerns among other stakeholders. Table 1 shows a list of planned PrEP trials in 2004 and 2005, including four trials terminated before or after enrollment had started.

**Table 1:** First generation of HIV pre-exposure prophylaxis trials

Sponsor	Place, expected start date	Population	Exposure	Sample size	Study aim	Duration (months)	Status
NIH/FHI	Cambodia, 2004	Women	Vaginal	960	Safety and efficacy	12	Stopped before start
FHI	Ghana, 2005	Women	Vaginal	400	Safety	12	Completed
FHI	Nigeria, 2005	Women	Vaginal	400	Safety	12	Stopped after enrolling 120
FHI	Cameroon, 2005	Women	Vaginal	400	Safety	12	Stopped after enrolling 400
FHI	Malawi, 2005	Heterosexual men	Penile	400	Safety	12	Stopped before start
CDC	Thailand, 2005	PWID	Parenteral	1200	Safety and efficacy	12	Completed
CDC	Botswana, 2005	Heterosexual men and women	Vaginal/penile	1600	Safety and efficacy	18	Completed
CDC	San Francisco, Atlanta, Boston, USA; 2005	MSM	Penile/rectal	400	Safety	15	Completed
NIH	Peru/Ecuador, 2007	MSM	Penile/rectal	1400	Safety and efficacy	18	Completed

NIH, National Institutes of Health; FHI360, Family Health International; CDC, US Center for Disease Control, PWID, people who inject drugs; MSM, men who have sex with men.

Four trials planned for implementation in Cambodia, Cameroon, Nigeria, and Malawi were cancelled due to controversies. The Cambodia trial, focused on PrEP safety and efficacy among female sex workers, never started due to concerns that, if women became HIV-infected, they would not have access to lifelong treatment, while losing their source of livelihood [35, 39–42]. In early 2005, similar studies in women at high HIV risk were cancelled in Cameroon (due to allegations of inappropriate standards [43]) and Nigeria (where the sponsor considered that conditions for study conduct were inappropriate [35, 37, 44]). In Malawi, the government stopped the trial due to fear of drug resistance developing if participants became infected during implementation [44]. The trial planned among people who injected drugs (PWID) in Thailand risked cancellation due to stakeholders’ allegations of unethical standards (i.e. not offering clean needles and syringes, in addition to methadone, according to WHO guidelines) [45]. The trial finished in May 2011 [12], but PWID organizations have reiterated that the trial was unethical and its conclusions are not acceptable [46].

In May 2005, a stakeholder consultation was convened with the participation of trial funders and community representatives from Cameroon, Ghana, Malawi, and Thailand [47]. Participants agreed that an immediate evaluation of trial design and protocol procedures was needed in ongoing and future trials to ensure compliance with the highest standards of care, civil society participation in trial design and conduct, and availability of mechanisms for feedback and conflict resolution at study sites [44]. Addressing one of the recommendations, the Joint United Nations Programme on HIV/AIDS, in collaboration with Global Advocacy for HIV Prevention (AVAC), consulting with communities, revised and produced a series of guidelines [48–52] for ethical assessment and definition of appropriate standards of study conduct in international HIV research. It conveyed the message that the scientific community had addressed the concerns raised about the ongoing trials, and that, together with communities, a new framework of operation had been established.

### Implementation of the major oral PrEP efficacy trials: mixed results and lessons learned

Increasing global access to ARTs and better-tolerated single and combined formulations, together with resolution of the initial controversies, eventually resulted in the implementation of PrEP trials. Some of the delayed PrEP trials, along with new ones, were rolled out between 2007 and 2013. Those trials included the following: two among women only (i.e. FEM-PrEP [53] and VOICE [54]), in addition to a vaginal gel study (CAPRISA 004 [9]); two among heterosexuals (i.e. Partners PrEP in serodiscordant couples and TDF2 in heterosexuals at high risk [12, 55]); one among men who have sex with men (MSM) and transwomen (iPrEx) in the Americas, South Africa, and Thailand [56]; and one among PWID (the CDC BTS) in Thailand [13]. Given concerns for resistance based on results of a preclinical study in macaques [57], the investigators of iPrEx and TDF2 decided to use, instead of tenofovir alone, the combination of tenofovir and emtricitabine (as offered in the formulation of Truvada^®^ by Gilead); while Partners PrEP opted to have separate arms for TDF and tenofovir - emtricitabine (TDF-FTC) for comparison. Table 2 shows the list of oral PrEP trials conducted, as well as their mixed results.

**Table 2:** PrEP randomized controlled trials and their findings

Study (reference)	Location	Population	Efficacy	

			Point estimate (%)	95% CI
iPrEx (Grant *et al*. 2010)	Peru, Ecuador, Brazil, United States, South Africa, Thailand	MSM	42	18 to 60%
Partners PrEP (Baeten *et al*. 2011)	Kenya, Uganda	Men	84	49 to 94%
		Women	66	19 to 82%
TDF2 (Thigpen *et al*. 2012)	Botswana	Men	80	25 to 97%
		Women	49	22 to 81%
FEM-PrEP (Van Damme *et al*. 2012)	Kenya, Tanzania	Women	6	−52 to 42%
VOICE (Marazzo *et al*. 2013)	South Africa, Uganda Zimbabwe	Women	−4	−50 to 30%
The CDC BTS (Choopaya *et al*. 2013)	Thailand	PWID	49	10 to 72%
Ipergay (Molina *et al*. 2015)	France, Canada	MSM	86	39 to 98%
PROUD (McCormack *et al*. 2015)	United Kingdom	MSM	86	58 to 96%

The protective effects of the first three PrEP trials (Table 2) completed in 2010 to 2011 (i.e. iPrEx, Partners PrEP, and TDF2) [12, 55] generated optimism. The subsequent termination of FEM-PrEP and VOICE due to futility [53, 54], however, led to assessments of the potential sources of such variability. Data on ARV concentration in serum, plasma, PBMCs, and hair showed highly variable adherence within and across sites, which likely explained important differences between intent-to-treat findings and those controlling for effective dose exposure. Overall adherence was extremely low in FEM-PrEP and VOICE, explaining their outcomes [58], as there is no evidence of interference of oral contraceptives in the protective effect of oral PrEP. Retrospective analyses that used mathematical modelling on those data showed that efficacy was strongly associated with detectable drug in serum or tissues. High adherence was associated with over 99% protection in iPrEx [59, 60]. Importantly, these analyses also showed the presence of “forgiveness”: oral PrEP is probably protective with less than daily dosing (although with no less than four doses per week), and such forgiveness may be lower in women due to relatively lower concentrations in vaginal tissue, compared to rectal tissue, after the same oral dose [61].

### Oral PrEP and effectiveness from subsequent studies

Low adherence levels in the efficacy trials raised concerns about the feasibility of PrEP as a public health strategy. Nevertheless it was recognized that real-life adherence to a product of demonstrated effectiveness would probably be different from adherence in a placebo-controlled trial, where participants are told that intervention efficacy is still unclear and that half of them are receiving a placebo [62]. This effect was demonstrated by the open label extension of iPrEx [63], where high levels of adherence were reported, and PrEP reduced incidence among those who consistently took the medication. Likewise, in October 2014, the UK PROUD study of immediate versus delayed PrEP for MSM accessing services at UK sexual health clinics stopped the deferred treatment arm and offered PrEP to all participants, given the protection demonstrated in their ongoing pilot study [64]. Two weeks later, the French Ipergay trial of intermittent, pericoital PrEP terminated the placebo arm based on an interim analysis that showed adherence and “considerable efficacy” [65]. In February 2015, findings from both studies showed similar (86%) effectiveness in preventing HIV infection among MSM at increased risk, who overall showed high adherence. The overall picture is that MSM who are motivated to use PrEP can achieve sufficient adherence to have even greater reduction in HIV as compared to iPrEx findings [66].

In a different epidemic context (i.e. serodiscordant couples in generalized epidemic settings), the Partners Demonstration Project, an open label observational study of PrEP and early treatment in Kenya and Uganda, also showed an overall relative risk reduction of 96% in an interim analysis [67]. These results suggest the use of PrEP as a bridge in serodiscordant couples – whereby the HIV-negative partner takes PrEP for protection while waiting for the HIV-positive partner to start treatment and minimize viral load. Several demonstration projects are starting in other countries, many of which are focusing on female sex workers (FSWs), namely, in Benin, India, Kenya, Senegal, South Africa, and Zimbabwe. It is becoming clear that these demonstration projects will help design PrEP implementation plans as part of combination prevention in programmatic contexts.

### The post-trial context of PrEP: effective need, programmatic dilemmas, and social paradoxes

As of early mid-2015, evidence supporting PrEP effectiveness could justify more active scaling up. However, should PrEP ever become an important component of the global HIV response, several issues need to be tackled.

#### Concerns prior to trial outcomes

Some early concerns have not been fully addressed or have adopted new dimensions. First, PrEP raised substantial resistance because it destabilized the social norm of “100% condom use,” which prevented so many infections in three decades, while in fact that social norm had already started to recede [68, 69]. Second, PrEP was misunderstood as intended to replace condoms, while in fact it was meant to become one element (but never the sole element) of the emerging paradigm of combination prevention [23]. Third, many objected to a perceived medicalization of HIV prevention, although this perception can be interpreted as fear of turning prevention into a mechanical process with no social-structural component; recent studies have shown that successful PrEP implementation retains the need for social interaction and the importance of community buy-in. Fourth, the controversies surrounding the early phases of the international PrEP trials led to fears that PrEP would be implemented in a compulsory way among key populations (e.g. sex workers) or serve as an excuse to not provide basic prevention tools (e.g. harm reduction for injection drug users), paying no attention to human rights [70–74]. From reactions so far, because PrEP is not cheap, its compulsory use seems unlikely with key populations anywhere. Fifth, PLH organizations and some policy makers have feared competition with treatment in an era of decreased resources, although it now seems clear that the PrEP component of HIV response, in order to remain cost-effective, should be focused on small fractions of the population at very high risk, while different strategies should be used with others [71]. Finally, PrEP may have generated a “moral panic” in certain stakeholders concerned about a potential loss of sexual restraints, leading to so-called risk compensation (i.e. having riskier sex and thereby neutralizing the benefit of PrEP). Even within the gay community, this concern has created a certain stigma affecting PrEP [75]. With the current media focus on PrEP and MSM, many assume that MSM should “be responsible and just use condoms,” which provide sufficient protection to them. This view fails to take into account the following: 1) for many MSM, condoms are not a feasible option, for several reasons, including loss of pleasure and power dynamics in relationships [76, 77]; 2) a more nuanced discussion is missing about the potential benefits of PrEP for women, including female sex workers and transwomen, for whom PrEP offers a prevention strategy that is under their control [78–80].

#### Public health and clinical guidance

In 2012, through its standard guideline development procedure, the WHO issued a conditional recommendation for PrEP use among serodiscordant couples and among men and transgender women who have sex with men, from a public health approach. It called for demonstration projects to assess conditions for potential PrEP implementation [81]. In 2014, the WHO updated its guidance and released a strong recommendation for governments to consider adding PrEP components to their combination prevention strategies for MSM in countries with high disease burden in those populations [23]. In the United States, a prophylactic indication for TDF-FTC for PrEP was approved in 2012 [82], and in early 2014 the US Center for Disease Control consolidated the indication of PrEP for people at risk for HIV acquisition [83].

Besides public health guidance (relevant for public prevention programmes set up by countries), clinical guidance is necessary where it is also recognized that PrEP is not meant to be used for life. As with any other prophylaxis, PrEP makes sense during periods of high exposure, which rarely cover an individual's entire life. Pericoital regimes, such as the focus of the Ipergay study [84], may also play a role in transitioning out of PrEP. The context in which oral PrEP may be individually recommended to some people may present some commonalities with the context in which other drugs are currently used and recommended for the prevention of other diseases. For example, statins are used to prevent cardiovascular disease, as they are essentially safe, like ARV drugs, but can rarely cause serious toxicity [85].

#### Population focus

PrEP is recommended for those facing a genuinely high risk of acquiring HIV. Clearly the benefit to be obtained from PrEP depends on the incidence rate of HIV and this factor has to be balanced against the (small) risks of the medication. Although adverse events are infrequent among positives and negatives alike, the benefits of treatment for those living with HIV are very high, whereas the benefits of PrEP to those who are HIV-negative depend entirely on their chance of acquiring infection. Based on available incidence estimations and behavioural data, PrEP use could have a clear positive impact among MSM almost everywhere, among sex workers in many places, among young people in southern Africa, but also among serodiscordant couples and those trying to conceive, and other key populations in various settings (e.g. partners of migrant labourers or truck drivers) [23]. Sound programmatic targeting is crucial to avoid PrEP ending up being prescribed mostly to the “worried well” [86]. Concerns remain as to how to target persons who would benefit from PrEP in generalized epidemics (potentially based on geographies with the highest incidence).

*Access* is determined by specific prevention guidance from normative bodies, drug availability, and a financing regime (e.g. out of pocket, insurance, public health programme). Concerns have been raised about the control that the pharmaceutical industry might have over the prices of PrEP regimes – which, at least in higher income countries, could be difficult to sustain [87–90]. Not without challenges, PrEP is slowly starting to be prescribed to at-risk Americans financed by medical insurance companies, national public health programmes for the poor or disabled, or through the producer of TDF-FTC, Gilead, via a drug assistance programme for underinsured individuals [91]. Some programmes are making PrEP a part of combination prevention, such as New York City's MSM programme [92] or the DREAMS initiative recently announced by U.S. President's Emergency Plan for AIDS Relief (PEPFAR) for 10 priority countries to provide a package of interventions aimed at tackling HIV among adolescent girls and young women [93].

Recent developments in European Union PrEP trials may accelerate regulatory changes there, too. However, TDF-FTC is not only not licensed for use as a preventive measure elsewhere, it is not even available for treatment in a few countries. In many countries, drugs can be used “off-label,” but usually only for acute indications with uncertain diagnosis or for life-threatening situations with no effective standard treatment, which would not be the case for PrEP. Nonetheless, demonstration projects ongoing in Brazil, several African countries, Thailand, and Australia, may lead to local approvals of TDF-FTC use for this indication. Finally, pricing is tied up with commercial decisions based on market estimations and trade agreements and also expressed in drug packaging and marketing.

#### The role of condoms

Given concerns about so-called risk compensation, normative bodies have decided to continue to maintain that PrEP should be used together with condoms [23, 83, 94]. However, initial data from demonstration studies in MSM show that people who choose to take PrEP may, in fact, be those who report episodes of unprotected anal intercourse, and their reported PrEP adherence is already high, with no subsequent risk compensation or change from their present condom use [95]. Hence, at least among MSM, PrEP may become a choice among people at risk due to condomless anal sex, who feel that a daily pill may suit them better than condoms. Perhaps a compromise in PrEP messaging could include stating the following: 1) PrEP does not intend to replace condoms but to add to condom protection; 2) PrEP does not protect against bacterial STIs; 3) PrEP can become especially useful for those who have difficulties with consistent condom use, as long as it is taken as prescribed.

#### Delivery

A few delivery models for local adaptation should be coming out soon from ongoing demonstration projects. It is likely, however, that some people who could benefit most from PrEP are those who find it most difficult to come routinely to a health service. Delivery models should be developed that are appropriate for the populations being served, while simultaneously being “fit for purpose” (e.g. they need to be integrated into the more holistic healthcare needs of the population, able to provide reliable HIV testing services, linked to HIV treatment services, able to detect serious toxicity, and able to refer complex or worrying cases into the broader health system) [96, 97]. Demonstration projects are evaluating delivery models. For example, PROUD delivered PrEP through sexual health clinics with quarterly visits in the United Kingdom [84, 98], whereas demonstration projects among sex workers in Benin and in South Africa are setting models where PrEP might work as part of a combination prevention package of PrEP and treatment as prevention (TasP). In India, PrEP is being evaluated to determine whether it can be implemented among brothel-based and street-based sex workers’ health services; and in Zimbabwe it is being offered in the context of highway-based sex work. US demonstration projects are evaluating customized prevention packages for MSM and transwomen (TW), for example, some that may include PrEP, the “testing and linking” of young MSM of colour to sexual health services, and text-messaging intervention to improve adherence.

#### Adherence, resistance to ARV and secondary effects

Practitioners feared that adherence in real life would be low (as in various trials), leading to resistance to a complex drug inappropriately used in primary care [99, 100]. Nevertheless, open label studies have shown that, among people who perceive its need, adherence can be very high [101]. Adherence must be a central message to users, despite the forgiveness shown by studies so far [59], where participants generally adhered well [63]. Because PrEP is given to HIV-uninfected people, it cannot cause resistance unless the person first acquires HIV and then continues to take PrEP. That is why it is essential to build delivery systems that reliably check the HIV status of those wanting to take or continue to take PrEP and that avoid informal distribution channels. Mathematical models show that most resistance comes from PLH who are not fully adherent to treatment; hence, preventing new infections through the use of PrEP could reduce rather than exacerbate levels of resistance in a community. Finally, stakeholders also feared drug toxicities and secondary effects [102, 103], but the experience so far has shown that they remain at reasonably low levels [104–107].

## Conclusions: perspectives and challenges in 2015 and beyond

A number of studies in the pipeline may streamline ARV-based prevention options even further, including the Ipergay trial (pericoital oral PrEP among MSM; this trial recently dropped the placebo arm [65]); the Ring Study and ASPIRE (designed to determine whether a monthly vaginal ring that delivers dapivirine helps prevent HIV infection in women and is safe for long-term use) [108, 109]; and studies of ARVs with long half-lives, such as rilpivirine and cabotegravir, to be administered parenterally every eight to twelve weeks [110, 111]. Such approaches to PrEP delivery may eventually become more widely applicable than oral PrEP, but it will be several years before they are manufactured, licensed, available, and affordable.

Although individual oral PrEP prescription may become the only form of PrEP available in many places, governments may implement population-focused PrEP programmes for cost-effectiveness, considering costs, affordability, and financing. However, decisions on focused PrEP programmes for populations with uncontrolled ongoing HIV transmission should preferably be based on impact. Given the high price of Truvada^TM^ in high income countries, PrEP programmatic feasibility will in part be defined by the pharmaceutical industry's role in providing access to supplies of TDF or TDF-FTC globally in the near future. The ongoing licensing and pricing of TDF-FTC in many places will be challenging, particularly in the context of new free-trade agreements [112]. Resolving current problems in treatment distribution in many countries and committing to ensuring its supply alongside the start of PrEP programmes will be central [113].

In HIV epidemics concentrated on MSM, self-selection of high-risk men insufficiently protected by condoms, with higher adherence to PrEP and no evidence of risk compensation, as observed in demonstration projects, suggests a desirable fit between a new tool and a population in need. However, it also demonstrates the importance of interdisciplinary studies and critical policy analysis to better understand how PrEP is actually adopted by at-risk communities and, under those conditions, to understand the following: what factors could improve or affect its effectiveness; how different forms of PrEP delivery would avert new infections and what the cost-effectiveness ratio would be; what role mathematical modelling could play in effectiveness and cost-effectiveness analysis; and how policy dialogue could be promoted to ensure that this strategy is considered fairly by governments.

In conclusion, over the years, important research findings have improved our understanding of biomedical and social determinants of HIV transmission. These findings have provided the evidence needed to transform the preventive response with the inclusion of “highly active” prevention approaches, as well as social and structural strategies at various levels, in what is now called a “combination prevention framework.” Among those prevention approaches, oral PrEP using TDF or TDF-FTC has emerged as an evidence-based option for people at risk of acquiring HIV. Despite its substantial potential, appropriate contribution of PrEP to the HIV response implies tackling two kinds of challenges: first, to clarify the numerous misconceptions that have led many to ignore the growing evidence of PrEP's utility; second, to fill research gaps concerning PrEP and its implementation and to resolve a number of issues related to its pertinence in different geographic and epidemiological contexts, health system structures and procedures, access, cost, and appropriate prioritization strategies. These are major challenges, proportional to the magnitude of the change we are witnessing in the dominant HIV prevention paradigm, one in which the impact of PrEP may finally help solidify the foundation of the so-far elusive concept of combination HIV prevention.

## References

[1] Kaufman MR, Cornish F, Zimmerman RS, Johnson BT (2014). Health behavior change models for HIV prevention and AIDS care: practical recommendations for a multi-level approach. J Acquir Immune Defic Syndr.

[2] Lettenmaier C, Kraft JM, Raisanen K, Serlemitsos E (2014). HIV communication capacity strengthening: a critical review. J Acquir Immune Defic Syndr.

[3] Detels R, Muñoz A, McFarlane G, Kingsley LA, Margolick JB, Giorgi J (1998). Effectiveness of potent antiretroviral therapy on time to AIDS and death in men with known HIV infection duration. Multicenter AIDS Cohort Study Investigators. JAMA.

[4] WHO, IATT, UNICEF (2013). Expanding and simplifying treatment for pregnant women living with HIV.

[5] Coates TJ, Richter L, Cáceres C (2008). Behavioural strategies to reduce HIV transmission: how to make them work better. Lancet.

[6] Auvert B, Taljaard D, Lagarde E, Sobngwi-Tambekou J, Sitta R, Puren A (2005). Randomized, controlled intervention trial of male circumcision for reduction of HIV infection risk: the ANRS 1265 trial. PLoS Med.

[7] Bailey RC, Moses S, Parker CB, Agot K, Maclean I, Krieger JN (2007). Male circumcision for HIV prevention in young men in Kisumu, Kenya: a randomised controlled trial. Lancet.

[8] Gray RH, Kigozi G, Serwadda D, Makumbi F, Watya S, Nalugoda F (2007). Male circumcision for HIV prevention in men in Rakai, Uganda: a randomised trial. Lancet.

[9] Abdool Karim Q, Abdool Karim SS, Frohlich JA, Grobler AC, Baxter C, Mansoor LE (2010). Effectiveness and safety of tenofovir gel, an antiretroviral microbicide, for the prevention of HIV infection in women. Science.

[10] Baeten JM, Donnell D, Ndase P, Mugo NR, Campbell JD, Wangisi J (2012). Antiretroviral prophylaxis for HIV prevention in heterosexual men and women. N Engl J Med.

[11] Grant R, Lama J, Anderson P, McMahan V, Liu A, Vargas L (2010). Preexposure chemoprophylaxis for HIV prevention in men who have sex with men. N Engl J Med.

[12] Thigpen MC, Kebaabetswe PM, Paxton LA, Smith DK, Rose CE, Segolodi TM (2012). Antiretroviral preexposure prophylaxis for heterosexual HIV transmission in Botswana. N Engl J Med.

[13] Choopanya K, Martin M, Suntharasamai P, Sangkum U, Mock PA, Leethochawalit M (2013). Antiretroviral prophylaxis for HIV infection in injecting drug users in Bangkok, Thailand (the Bangkok Tenofovir Study): a randomised, double-blind, placebo-controlled phase 3 trial. Lancet.

[14] Boyles S (1995). STD treatment reduces HIV in rural Tanzania. AIDS Wkly.

[15] Cohen MS, Chen YQ, McCauley M, Gamble T, Hosseinipour MC, Kumarasamy N (2011). Prevention of HIV-1 infection with early antiretroviral therapy. N Engl J Med.

[16] Hoffman IF, Taha TE, Padian NS, Kelly CW, Welch JD, Martinson FE (2004). Nonoxynol-9 100 mg gel: multi-site safety study from sub-Saharan Africa. AIDS.

[17] Buchbinder S, Mehrotra D, Duerr A, Fitzgerald D, Mogg R, Li D (2008). Efficacy assessment of a cell-mediated immunity HIV-1 vaccine (the step study): a double-blind, randomised, placebo-controlled, test-of-concept trial. Lancet.

[18] WHO

[19] Buot ML, Docena JP, Ratemo BK, Bittner MJ, Burlew JT, Nuritdinov AR (2014). Beyond race and place: distal sociological determinants of HIV disparities. PLoS One.

[20] Hirsch JS (2014). Labor migration, externalities and ethics: theorizing the meso-level determinants of HIV vulnerability. Soc Sci Med.

[21] Auerbach JD, Parkhurst JO, Caceres CF (2011). Addressing social drivers of HIV/AIDS for the long-term response: conceptual and methodological considerations. Glob Public Health.

[22] Hankins CA, Dybul MR (2013). The promise of pre-exposure prophylaxis with antiretroviral drugs to prevent HIV transmission. Curr Opin HIV AIDS.

[23] WHO, editor (2014). Policy brief: HIV prevention, diagnosis, treatment and care for key populations.

[24] Padian NS, Mccoy SI, Manian S, Wilson D, Schwartländer B, Bertozzi SM (2011). Evaluation of large-scale combination HIV prevention programs: essential issues. J Acquir Immune Defic Syndr.

[25] Biamonte MA, Wanner J, Le Roch KG (2013). Recent advances in malaria drug discovery. Bioorg Med Chem Lett.

[26] Upke IS, Moonasar D, Raman J, Barnes KI, Baker L, Blumberg L (2013). Case management of malaria: treatment and chemoprophylaxis. S Afr Med.

[27] Feldstein CA (2008). Statins as antihypertensives. Recent Pat Cardiovasc Drug Discov.

[28] Simko F, Pechanova O (2009). Potential roles of melatonin and chronotherapy among the new trends in hypertension treatment. J Pineal Res.

[29] Sepanlou SG, Farzadfar F, Jafari E, Danaei G (2012). Cardiovascular disease prevention using fixed dose pharmacotherapy in Iran: updated meta-analyses and mortality estimation. Arch Iran Med.

[30] Steyn DW, Steyn P (2007). Low-dose dopamine for women with severe pre-eclampsia. Cochrane Database Syst Rev.

[31] Genest DS, Falcao S, Michel C, Kajla S, Germano MF, Lacasse AA (2013). Novel role of the renin-angiotensin system in preeclampsia superimposed on chronic hypertension and the effects of exercise in a mouse model. Hypertension.

[32] Dorniak-Wall T, Grivell RM, Dekker GA, Hague W, Dodd JM (2014). The role of L-arginine in the prevention and treatment of pre-eclampsia: a systematic review of randomised trials. J Hum Hypertens.

[33] Podzamczer D, Santin M, Jimenez J, Casanova A, Bolao F, Gudiol GR (1993). Thrice-weekly cotrimoxazole is better than weekly dapsone-pyrimethamine for the primary prevention of Pneumocystis carinii pneumonia in HIV-infected patients. AIDS.

[34] WHO Consolidated guidelines on the use of antiretroviral drugs for treating and preventing HIV infection.

[35] Singh JA, Mills EJ (2005). The abandoned trials of pre-exposure prophylaxis for HIV: what went wrong?. PLoS Med.

[36] Grohskopf LA, Chillag KL, Gvetadze R, Liu AY, Thompson M, Mayer KH (2013). Randomized trial of clinical safety of daily oral tenofovir disoproxil fumarate among HIV-uninfected men who have sex with men in the United States. J Acquir Immune Defic Syndr.

[37] Peterson L, Taylor D, Clarke E, Doh A, Phillips P, Belai G (2006). Findings from a double-blind, randomized placebo-controlled trial of tenofovir disoproxil fumarate (TDF) for prevention of HIV infection in women.

[38] Mayer KH, Karim SA, Kelly C, Maslankowski L, Rees H, Profy AT (2003). Safety and tolerability of vaginal PRO 2000 gel in sexually active HIV-uninfected and abstinent HIV-infected women. AIDS.

[39] Lancet T (2005). The trials of tenofovir trials. Lancet.

[40] Page-Shafer K, Saphonn V, Penh Sun L, Chi vun M, Cooper DA, Kaldor JM (2005). HIV prevention research in resource-limited setting: the experience of planning a trial in Cambodia. Lancet.

[41] Cohen J (2005). More woes for novel HIV prevention approach. Science.

[42] Loff B, Jenkins C, Ditmore M, Overs C, Barbero R (2005). Unethical clinical trials in Thailand: a community response. Lancet.

[43] Mills E, Rachlis B, Wu P, Wong E, Wilson K, Singh S (2005). Media reporting of tenofovir trials in Cambodia and Cameroon. BMC Int Health Hum Rights.

[44] Cairns G, Alcorn K, Bernard EJ, Carter M, Smart T (2006). Preventing HIV.

[45] Chua A, Ford N, Wilson D, Cawthorne P (2005). The tenofovir pre-exposure prophylaxis trial in Thailand: researchers should show more openness in their engagement with the community. PLoS Med.

[46] AIDSMEDS (2013). Trial of PrEP in drug users called into question [Internet].

[47] IAS (2005). Stakeholder consultation to address issues related to tenofovir prophylactic research.

[48] UNAIDS (2000). Ethical considerations in HIV preventive vaccine research.

[49] UNAIDS (2012). Ethical considerationsin biomedical HIV prevention trials.

[50] UNAIDS (2007). Good participatory practice: guidelines for biomedical HIV prevention trials.

[51] UNAIDS (2011). Good participatory practice: guidelines for biomedical HIV prevention trials.

[52] UNAIDS (2004). Ethical considerations in HIV preventive vaccine research.

[53] Van Damme L, Corneli A, Ahmed K, Agot K, Lombaard J, Kapiga S (2012). Preexposure prophylaxis for HIV infection among African women. N Engl J Med.

[54] Marrazzo JM, Ramjee G, Richardson BA, Gomez K, Mgodi N, Nair G (2015). Tenofovir-based preexposure prophylaxis for HIV infection among African women. N Engl J Med.

[55] Baeten JM, Donnell D, Ndase P, Mugo NR, Campbell JD, Wangisi J (2012). Antiretroviral prophylaxis for HIV prevention in heterosexual men and women. N Engl J Med.

[56] Grant RM, Lama JR, Anderson PL, McMahan V, Liu AY, Vargas L (2010). Preexposure chemoprophylaxis for HIV prevention in men who have sex with men – supplemental appendix. N Engl J Med.

[57] Garcia-Lerma JG, Otten RA, Qari SH, Jackson E, Cong ME, Masciotra S (2008). Prevention of rectal SHIV transmission in macaques by daily or intermittent prophylaxis with emtricitabine and tenofovir. PLoS Med.

[58] Murnane PM, Heffron R, Ronald A, Bukusi EA, Donnell D, Mugo NR (2014). Pre-exposure prophylaxis for HIV-1 prevention does not diminish the pregnancy prevention effectiveness of hormonal contraception. AIDS.

[59] Anderson PL, Glidden DV, Liu A, Buchbinder S, Lama JR, Guanira JV (2012). Emtricitabine-tenofovir concentrations and pre-exposure prophylaxis efficacy in men who have sex with men. Sci Transl Med.

[60] Donnell D, Baeten JM, Bumpus NN, Brantley J, Bangsberg DR, Haberer JE (2014). HIV Protective efficacy and correlates of tenofovir blood concentrations in a clinical trial of PrEP for HIV prevention. J Acquir Immune Defic Syndr.

[61] Cortrell ML, Yang KH, Prince HMA, Sykes C, White N, Malone S (2014). Predicting effective Truvada PrEP dosing strategies with a novel PK-PD model incorporating tissue active metabolites and endogenous nuclootides (EN).

[62] Amico KR, Stirratt MJ (2014). Adherence to preexposure prophylaxis: current, emerging, and anticipated bases of evidence. Clin Infect Dis.

[63] Grant RM, Anderson PL, McMahan V, Liu A, Amico KR, Mehrotra M (2014). Uptake of pre-exposure prophylaxis, sexual practices, and HIV incidence in men and transgender women who have sex with men: a cohort study. Lancet Infect Dis.

[64] Medical Research Council Press Office (2014). PROUD study interim analysis finds pre-exposure prophylaxis (PrEP) is highly protective against HIV for gay men and other men who have sex with men in the UK [press release].

[65] ANRS UN grand succes dans la lutte contre le vih/SIDA. Un médicament pris au moment des rapports sexuels réduit efficacement le risque d'infection [Internet]. ANRS.

[66] Cohen S, Vittinghoff E, Anderson P, Doblecki-Lewis S, Bacon O, Chege W (2014). Implementation of PrEP in STD clinics: high uptake and drug detection among MSM in the demonstration project.

[67] Baeten J (2015). Near elimination of HIV transmission in a demonstration project of PrEP and ART.

[68] Guest G, Shattuck D, Johnson L, Akumatey B, Clarke EE, Chen PL (2008). Changes in sexual risk behavior among participants in a PrEP HIV prevention trial. Sex Transm Dis.

[69] Paxton LA (2012). Considerations regarding antiretroviral chemoprophylaxis and heterosexuals in generalized epidemic settings. Curr Opin HIV AIDS.

[70] O'Hara KM (2012). Pre-exposure prophylaxis: where HIV prevention and responsibility intersect. JAAPA.

[71] Bailey TC, Sugarman J (2013). Social justice and HIV vaccine research in the age of pre-exposure prophylaxis and treatment as prevention. Curr HIV Res.

[72] Philpott S (2013). Social justice, public health ethics, and the use of HIV pre-exposure prophylaxis. Am J Prev Med 1.

[73] Rowniak S, Portillo C (2013). Pre-exposure prophylaxis: an ethical discussion. J Assoc Nurses AIDS Care.

[74] Venter F, Allais L, Richter M (2014). Exposure ethics: does HIV pre-exposure prophylaxis raise ethical problems for the health care provider and policy maker?. Bioethics.

[75] Brooks RA, Landovitz RJ, Kaplan RL, Lieber E, Lee SJ, Barkley TW (2012). Sexual risk behaviors and acceptability of HIV pre-exposure prophylaxis among HIV-negative gay and bisexual men in serodiscordant relationships: a mixed methods study. AIDS Patient Care STDS.

[76] Johnson BT, Scott-Sheldon LA, Smoak ND, Lacroix JM, Anderson JR, Carey MP (2009). Behavioral interventions for African Americans to reduce sexual risk of HIV: a meta-analysis of randomized controlled trials. J Acquir Immune Defic Syndr.

[77] Albarracin D, Durantini MR (2010). Are we going to close social gaps in HIV? Likely effects of behavioral HIV-prevention interventions on health disparities. Psychol Health Med.

[78] Peng B, Yang X, Zhang Y, Dai J, Liang H, Zou Y (2012). Willingness to use pre-exposure prophylaxis for HIV prevention among female sex workers: a cross-sectional study in China. HIV/AIDS.

[79] Jackson T, Huang A, Chen H, Gao X, Zhang Y, Zhong X (2013). Predictors of willingness to use HIV pre-exposure prophylaxis among female sex workers in Southwest China. AIDS Care.

[80] Mack N, Evens EM, Tolley EE, Brelsford K, Mackenzie C, Milford C (2014). The importance of choice in the rollout of ARV-based prevention to user groups in Kenya and South Africa: a qualitative study. J Int AIDS Soc.

[81] WHO (2012). Guidance on pre-exposure oral prophylaxis (prep) for serodiscordant couples, men and transgender women who have sex with men at high risk of HIV: recommendations for use in the context of demonstration projects.

[82] FDA (2012). FDA approves first drug for reducing the risk of sexually acquired HIV infection.

[83] CDC (2014). Pre exposure prophylaxis for the prevention of HIV infection in the United States/2014. Guidelines.

[84] Molina J-M (2015). On demand PrEP with oral TDF-FTC in MSM: results of the ANRS Ipergay trial.

[85] D'Agostino RB, Ansell BJ, Mora S, Krumholz HM (2014). Clinical decisions. The guidelines battle on starting statins. N Engl J Med.

[86] Mera R, Ng LK, Magnuson D, Campos A, Silva M, Rawling M (2014). Characteristics of Truvada for pre-exposure prophylaxis users in the US (January 2012–September 2013). HIV Drug Therapy in the Americas.

[87] Gomez GB, Borquez A, Case KK, Wheelock A, Vassall A, Hankins C (2013). The cost and impact of scaling up pre-exposure prophylaxis for HIV prevention: a systematic review of cost-effectiveness modelling studies. PLoS Med.

[88] Hellinger FJ (2013). Assessing the cost effectiveness of pre-exposure prophylaxis for HIV prevention in the US. Pharmacoeconomics.

[89] Verguet S, Stalcup M, Walsh JA (2013). Where to deploy pre-exposure prophylaxis (PrEP) in sub-Saharan Africa?. Sex Transm Infect.

[90] Alistar SS, Grant PM, Bendavid E (2014). Comparative effectiveness and cost-effectiveness of antiretroviral therapy and pre-exposure prophylaxis for HIV prevention in South Africa. BMC Med.

[91] Flash C, Landovitz R, Mera Giler R, Ng L, Magnuson D, Bush Wooley S (2014). Two years of Truvada for pre-exposure prophylaxis utilization in the US. Glasgow: International Congress of Drug Therapy in HIV Infection.

[92] (2015). New York City: Department of Health and Mental Hygiene. Health Department Lounches New PrEP and PEP Campaign: new ways to prevent HIV [Press release].

[93] PEPFAR (2014). The U.S. President's emergency plan for AIDS relief, the Bill & Melinda Gates Foundation, and the Nike Foundation Partner on $210 million initiative to reduce new HIV infections in adolescent girls and young women [Internet].

[94] CONSENSUS COMMITTEE (2012). Southern African guidelines for the safe use of pre-exposure prophylaxis in men who have sex with men who are at risk for HIV infection. Southern African Journal of HIV Medicine.

[95] Marcus JL, Glidden DV, Mayer KH, Liu AY, Buchbinder SP, Amico KR (2013). No evidence of sexual risk compensation in the iPrEx trial of daily oral HIV preexposure prophylaxis. PLoS One.

[96] Pines HA, Gorbach PM, Weiss RE, Shoptaw S, Landovitz RJ, Javanbakht M (2014). Sexual risk trajectories among MSM in the United States: implications for pre-exposure prophylaxis delivery. J Acquir Immune Defic Syndr.

[97] Boffito M, Jackson A, Owen A, Becker S (2014). New approaches to antiretroviral drug delivery: challenges and opportunities associated with the use of long-acting injectable agents. Drugs.

[98] McCormack S (2015). Programatic open-label randomised trial of preesposure prophylaxis: the PROUD study.

[99] Tellalian D, Maznavi K, Bredeek UF, Hardy WD (2013). Pre-exposure prophylaxis (PrEP) for HIV infection: results of a survey of HIV healthcare providers evaluating their knowledge, attitudes, and prescribing practices. AIDS Pat Care STDS.

[100] Gengiah TN, Moosa A, Naidoo A, Mansoor LE (2014). Adherence challenges with drugs for pre-exposure prophylaxis to prevent HIV infection. Int J Clin Pharm.

[101] Grant RM (2015). Scale-up of preesposure prophylaxis in San Francisco to impact HIV incidence.

[102] Ware NC, Wyatt MA, Haberer JE, Baeten JM, Kintu A, Psaros C (2012). What's love got to do with it? Explaining adherence to oral antiretroviral pre-exposure prophylaxis for HIV-serodiscordant couples. J Acquir Immune Defic Syndr.

[103] Wade Taylor S, Mayer KH, Elsesser SM, Mimiaga MJ, O'Cleirigh C, Safren SA (2014). Optimizing content for pre-exposure prophylaxis (PrEP) counseling for men who have sex with men: perspectives of PrEP users and high-risk PrEP naive men. AIDS Behav.

[104] Abraham BK, Gulick R (2012). Next-generation oral preexposure prophylaxis: beyond tenofovir. Curr Opin HIV AIDS.

[105] Jespers V, Millwood IY, Poynten IM, Van Damme L, Kaldor JM (2013). The evolving design and methods for trials evaluating the safety of candidate vaginal microbicides: a systematic review. Sex Transm Dis.

[106] Krakower D, Mayer KH (2012). What primary care providers need to know about preexposure prophylaxis for HIV prevention: a narrative review. Ann Intern Med.

[107] Mayer KH (2014). Antiretroviral chemoprophylaxis: state of evidence and the research agenda. Clin Infect Dis.

[108] ClinicalTrials.gov (2014). Safety and effectiveness of tenofovir gel in the prevention of human immunodeficiency virus (HIV-1) infection in women and the effects of tenofovir gel on the incidence of herpes simplex virus (HSV-2) infection US [Internet].

[109] ClinicalTrials.gov (2014). Project ASPIRE efficacy pilot: achieving superior parental involvement for rehabilitative excellence US [Internet].

[110] ClinicalTrials.gov (2014). A phase IIb study to evaluate a long-acting intramuscular regimen for maintenance of virologic suppression (following induction with an oral regimen of GSK1265744 and Abacavir/Lamivudine) in human immunodeficiency virus type 1 (HIV-1) infected, antiretroviral therapy-naive adult subjects US [Internet].

[111] Spreen W, Ford SL, Chen S, Wilfret D, Margolis D, Gould E (2014). GSK1265744 pharmacokinetics in plasma and tissue following single-dose long-acting (LA) injectable administration in healthy subjects. J Acquir Immune Defic Syndr.

[112] Westerhaus M, Castro A (2006). How do intellectual property law and international trade agreements affect access to antiretroviral therapy?. PLoS Med.

[113] Windisch R, Waiswa P, Neuhann F, Scheibe F, de Savigny D (2011). Scaling up antiretroviral therapy in Uganda: using supply chain management to appraise health systems strengthening. Global Health.

